# Numb Chin Syndrome – a reflection of systemic malignancy

**DOI:** 10.1186/1477-7819-4-52

**Published:** 2006-08-09

**Authors:** Ramesh Kapa Baskaran, Krishnamoorthy, Mark Smith

**Affiliations:** 1 Department of General Surgery, Royal Lancaster Infirmary, Lancaster LA1 4RP, UK; 2 Department of Anaesthesia, Royal Lancaster Infirmary, Lancaster LA1 4RP, UK; 3 Department of Intensive Care, Royal Lancaster Infirmary, Lancaster LA1 4RP, UK

## Abstract

**Background:**

Numb chin is an uncommon underappreciated though well documented neurological manifestation of metastatic malignancy.

**Case presentation:**

A 50-year-old patient presented with right numb chin for few days. No dental cause was found. He later developed generalized vague symptoms. In few weeks his liver and renal functions deteriorated. Blood picture showed leucoerythroblastic picture. CT scan and bone marrow biopsy done at this stage revealed underlying high grade lymphoblastic lymphoma. He went into multiorgan failure, requiring ventilatory support and death within a short period.

**Conclusion:**

Numb Chin is a syndrome which initially presents with unilateral numbness or orofacial pain in the distribution of the inferior alveolar nerve and its branches with an underlying systemic malignancy. We emphasise that physicians and dentists should consider metastatic cancer in any patient who presents with chin or jaw numbness where no other obvious cause for their complaint is found.

## Background

Numb Chin Syndrome (NCS) is characterised by hypoesthesia, par aesthesia or pain over the chin in the region supplied by mental nerve and its branches [1,2]. This is a pure sensory neuropathy because the inferior alveolar nerve has no motor fibres. The chin numbness is caused by malignant infiltration of the inferior alveolar nerve sheath or compression of the nerve by jaw metastases or local tumour [3]. Intracranial involvement of the mandibular nerve by lesion at the level of base of skull is also reported [4]. This syndrome is most often a forerunner to malignancy progression and relapse [4-6]. We describes a case of NCS caused by high-grade lymphocytic lymphoma. We would like to highlight this rare and unusual condition as considering it as a differential diagnosis may help in early intervention and improve the prognosis. The clinical course and rapid deterioration since the initial presentation of this syndrome in a patient is discussed.

## Case presentation

A 50-year-old man presented to his dentist with pain over right side of chin and numbness for a few days and was treated for a dental abscess on clinical suspicion, though X-ray appeared normal and it was a dry tap on drilling, which excludes dental abscess as the cause of his symptom.

Two weeks later he was admitted to Accident and Emergency (A & E) department following generalised muscle ache, fatigue, weakness, anorexia, nausea, vomiting and sweating for more than 24 hours. He was treated with intravenous fluids, antibiotics and analgesics and discharged form A & E with a diagnosis of infection following dental intervention.10 days later this gentleman returned with complaint of shock like sensation in lower lips and a numb chin and was admitted in medical ward.

His past medical history was of diet controlled diabetes mellitus, well controlled hypertension and hypothyroidism on regular thyroxine.

Examination revealed absent sensation to touch and pain in mandibular division of Trigeminal Cranial Nerve below the lower lip on right side. Other cranial nerves and central nervous system examination were normal. All other systems were found to be clinically normal.

Initial investigation showed an elevated C reactive protein (CRP)-139.2 mg/l and a slightly raised gamma glutamyl transferase-173 iu/l, normal full blood count, creatinine, urea and electrolytes, glucose, liver function test and chest X-ray at the time of initial presentation. The differential diagnosis at this stage was of a non-specific viral illness, atypical viral myositis or atypical Guillain-Barre syndrome.

Since the muscle ache, low grade fever persisted and the numbness of chin started to spread beyond the midline to other side a computerised tomography (CT) scan head was done which showed no focal lesions. Viral markers were negative and blood/urine culture showed no bacterial growth. Urine for Leptospirosis screen was also negative. Echocardiogram showed normal cardiac function and no evidence of bacterial endocarditis. Ultrasound abdomen was performed but poor views were found due to the patient's body habitus.

In the next 2 weeks the liver function test became deranged. CRP and erythrocyte sedimentation rate (ESR) remained elevated. Slight weakness of left lower limb compared to right was noted together with new onset of right axillary lymphadenopathy (5 × 5 cm). The patient was managed by antibiotics, pain killers, vitamins and nutrition. In the meantime the renal function started to deteriorate and the patient became oliguric with signs of fluid overload, increasing potassium, urea and creatinine. The blood film done at this stage showed a leucoerythroblastic picture and lymphocytosis. The patient was hypercalcemic (3.0 mmol/l). Anaerobic blood culture showed growth of gram positive cocci for which Teicoplanin was started. The patient was admitted to ICU (Intensive care unit) at this stage for supporting renal and hepatic function.

We suspected a lymphoproliferative disorder at this time and decided to do a CT scan thorax and abdomen, Bone Marrow biopsy and biopsy of axillary lymph node.

CT scan thorax revealed a large solitary right axillary node but no other mediastinal or abdominal lymphadenopathy. Lung fields were clear with mild bilateral pleural effusion. There was bilateral renal enlargement clinically due to acute nephritis.

The patient was supported by continuous veno venous hemofiltraion (CVVH) because of worsening renal function via right femoral venous access. Arterial blood gas (ABG) showed worsening acidosis in spite of CVVH. pH-7.315, pO_2_-117.9 mmHg, PCo_2_-31.7 mm Hg, BE-9.4, HCO_3_-15.8 mmol/L. Filter was changed to a large size and predilution fluid volume increased. In spite of changes in CVVH acidosis improved a little and hence was started on NaHco_3 _infusion of 100 ml/hour to a total of 300 ml.

**Figure 1:**
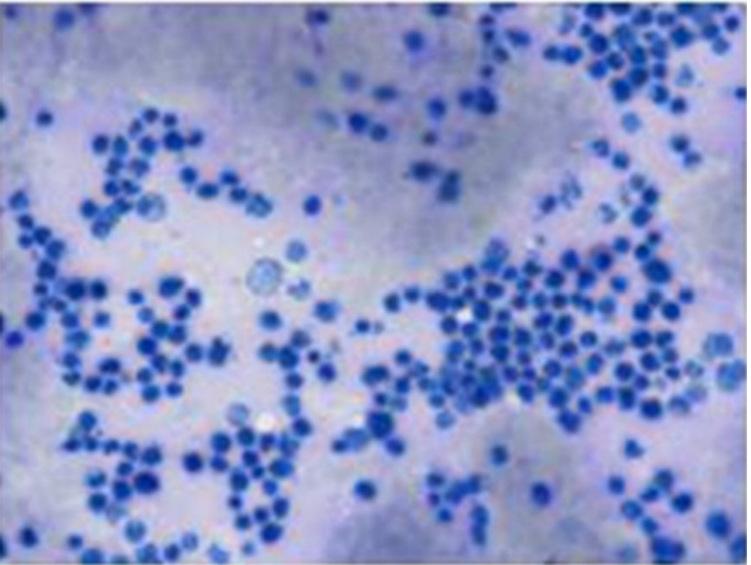
**Modified Wright's staining (100× magnification): **Hypercellular marrow showing 60–70% large lymphoblasts with marked vacuolated basophilic cytoplasm, by large extent diagnostic of Lymphoblastic Lymphoma.

**Figure 2:**
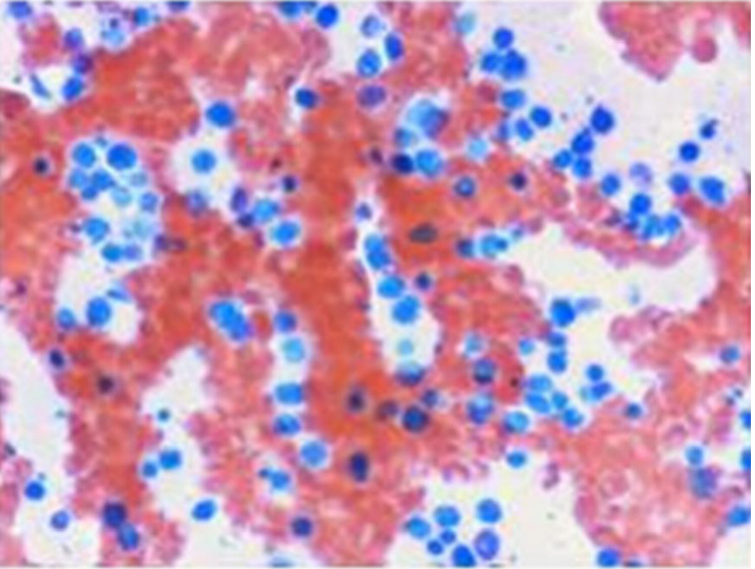
**PAS Stain (100× magnifications): **Negative.

Bone marrow aspirate showed 70% infiltration with lymphoblastic lymphoma very aggressive type and unlikely to get into remission (Figure 1 and 2).

Resistant worsening acidosis inspite of CVVH, increasing ventilator support, increasing requirement of adrenaline and nor adrenaline to maintain hemodynamic stability with a diagnosis of acute lymphoblastic lymphoma with multi organ failure, we decided to withdraw the support after discussing with family members. The patient died after few minutes of withdrawal of support. All these happening within a span of 6 weeks from his initial presentation with a numb chin.

## Discussion

Numb Chin Syndrome, mental nerve neuropathy is a sensory neuropathy presenting with numbness of the chin in the distribution of the mental nerve and its branches of the mandibular division of the trigeminal nerve. The causes include 1) Odontogenic-dental abscess, dental anaesthesia, dental trauma, osteomyelitis and benign tumours 2) Systemic-amyloidosis, sickle cell disease, sarcoidosis, multiple sclerosis, HIV, diabetes mellitus 3) Malignancy-Lymphoma, breast cancer, leukaemia, lung cancer, prostate cancer and head and neck cancers.

Though rare, it may be the first symptom and manifestation in the presentation of an underlying malignancy [7]. The most common primary malignancy being lymphoma or breast cancer that has metastasized to mandible [2,8]. Other symptoms include numbness over the lower lip, chin and gingival mucosa, but motor function of lower face remains intact [2,4,8]. Symmetrical involvement has been reported in 10% [2]. Mechanism of this syndrome in cancer patients may be one of the following: metastasis or invasion of mandible causing compression on the nerve, base of skull lesions, leptomeningeal seeding, perineural or neural invasion or paraneoplastic syndrome [3].

In the above discussed case the cause of NCS is infiltration of malignant cells onto the nerve sheath as X-ray and CT of skull was normal. Prognosis of NCS in the patient with cancer is poor and survival is usually measured in months.

## Conclusion

Though rare NCS should be one of the differential diagnosis dentists and clinicians must be aware of when a patient presents with numbness in chin. The trivial symptom of lower facial numbness may signal serious disease.

## Competing interests

The author(s) declare that they have no competing interests.

## Authors' contributions

**RKB **and **KM**: Acquisition of Data, Preparation of manuscript, design, revising and final submission.

**MS **helped reviewing and contributed to critical revisions.

All authors read and approved the final manuscript.
